# A droplet that releases stress: Drop‐printing for conformal bioelectronics at neural and soft‐tissue interfaces

**DOI:** 10.1002/ctm2.70659

**Published:** 2026-04-16

**Authors:** Qin Xu, Jiang Li, Xingyu Yang, Wenjianlong Zhou, Wang Jia

**Affiliations:** ^1^ Department of Neurosurgery, Beijing Tiantan Hospital, National Center for Neurological Disorders Capital Medical University Beijing China

## 1 WHEN PLACEMENT BECOMES THE BOTTLENECK

Bioelectronic interfaces are moving from proof‐of‐concept devices toward clinical tools for sensing, stimulation and closed‐loop therapy. Landmark examples such as high‐density flexible electrocorticography (ECoG) arrays and long‐term multimodal ‘electronic dura mater’ systems highlight the promise of tissue‐conformal neural interfaces—but also reveal a practical bottleneck: deploying film devices, typically fabricated on planar substrates, onto living, three‐dimensional (3D) anatomy.^1^, ^2^ A thin film that performs well on a flat wafer can fracture, wrinkle, or delaminate when wrapped onto curved, textured, and hydrated tissues. For clinicians, therefore, the key issue is not only whether such interfaces function electrically, but whether they can be positioned safely, reproducibly, and efficiently on patient‐specific anatomy within a wet, time‐constrained surgical field.^3^, ^4^

The central difficulty lies in stress accumulation during the conformal deformation of nonstretchable films. Wrapping onto 3D surfaces imposes localised stretching, bending, and compression, and once the resulting in‐plane strain exceeds the fracture limit, fragile metal‐ or silicon‐based components can fail. To mitigate this problem, the field has mainly pursued two strategies: redesigning nonstretchable materials into stretchable layouts, such as serpentine, mesh, or kirigami‐like structures, or replacing them with softer conductors and semiconductors.^5^, ^6^ Although effective, these approaches often introduce additional design and processing complexity and may compromise device performance. Inspired by the biological ‘slide’ mechanism, drop‐printing offers an alternative route: rather than forcing films to stretch, it enables conformal transfer through stress release during wrapping.^7^

## 2 A DROPLET ENABLES STRESS‐FREE DEVICE PLACEMENT

**FIGURE 1:**
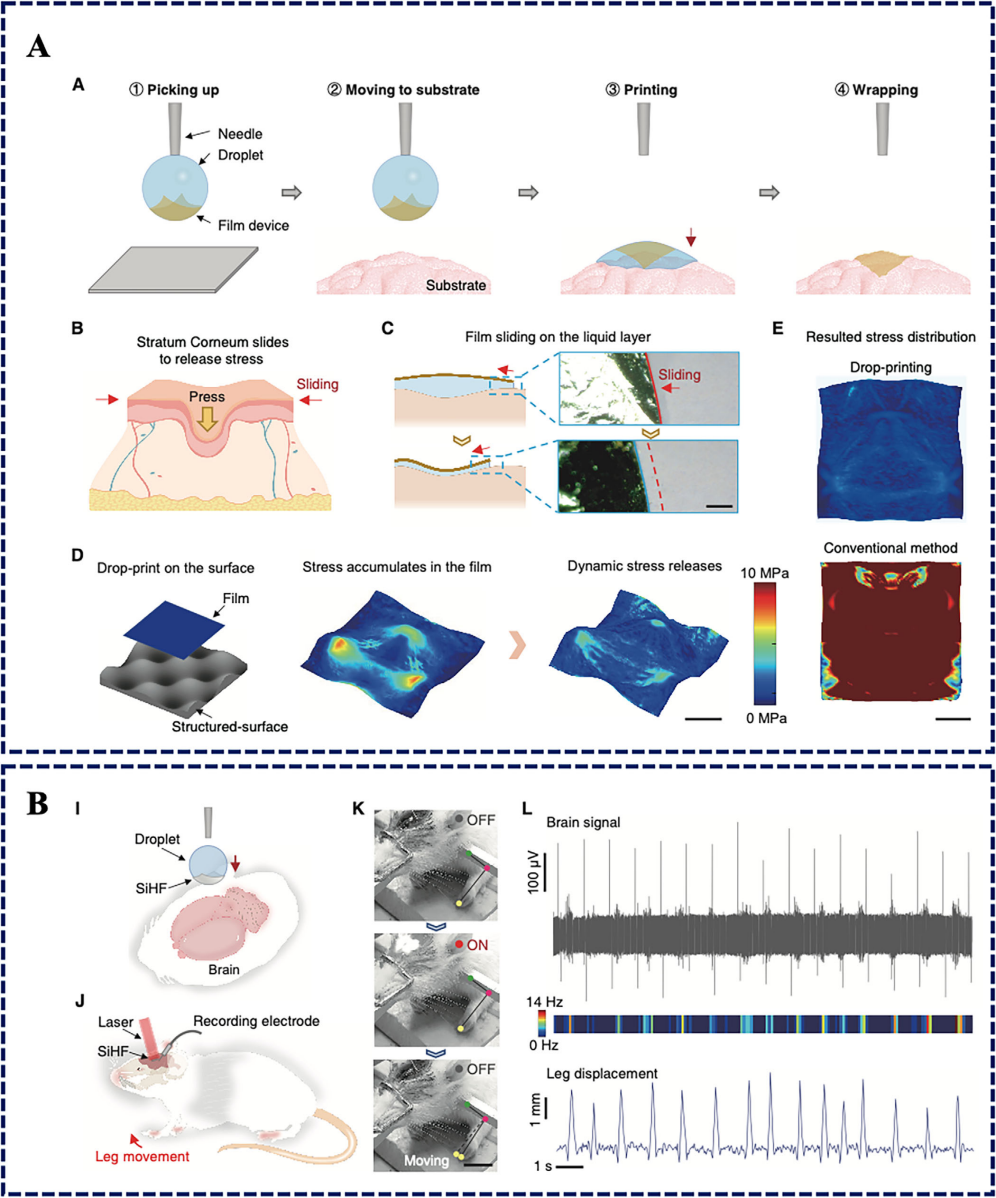
Drop‐printing with dynamic stress release for conformal bioelectronics and optoelectronic neuromodulation. (A) Schematic workflow of picking up, printing and wrapping a thin‐film device; the transient liquid layer enables local sliding to release stress during 2D‐to‐3D adaptation. (B) Proof‐of‐concept brain‐surface optoelectronic stimulation with a drop‐printed silicon heterojunction film, showing representative brain signals and limb movements. Adapted from Li et al.^7^

Drop‐printing uses a droplet to pick up, transport and release ultrathin films onto a target surface. Following deposition, the droplet forms a transient solid‐liquid interface that fulfils two clinically critical functions. First, capillary forces during spreading and evaporation drive conformal contact without strong external pressing. Second, the liquid layer lowers interfacial friction and permits local sliding, providing a practical route to release stress as the film adapts from 2D to 3D geometry. This matters because bending‐dominated deformation is far less damaging than in‐plane stretching in thin films; by allowing sliding and local wrinkling, drop‐printing limits stress concentration that otherwise breaks metal traces or fractures brittle semiconductors. The workflow and stress‐release mechanism are summarised in Figure 1.^7^

Conventional stamp‐based transfer can require newton‐level forces applied for seconds to force conformity—an approach that risks damaging fragile films and is undesirable on delicate tissues. In contrast, droplet impact delivers only a brief, millinewton‐scale impulse over milliseconds. This minimised mechanical input is highly advantageous for clinical translation, as both device integrity and tissue safety are closely linked to the deployment method used.^7^

## 3 WHY THIS MATTERS CLINICALLY

Clinical bioelectronics increasingly require spatial specificity: which fascicle is stimulated, which cortical region is mapped, and whether contact remains stable during micromotion. Drop‐printing adds placement control through the three‐phase contact line (TCL), which constrains film motion during evaporation. By tuning droplet geometry and wetting conditions, and by using trace additives that pin the TCL, positional deviations can be reduced to the micrometre scale for millimetre‐scale films—an accuracy level that can matter for stimulation thresholds, selectivity and signal quality.^3^, ^7^

A second advantage is ‘droplet programmability’. The droplet is not only a mechanical carrier but also a tuneable interfacial medium. Physiological solutions (e.g., PBS) support transfer onto biological tissues, and the same approach can move cell‐coated films while preserving cell distribution. Additives can further tune adhesion strength and environmental robustness, offering practical levers to adapt the method to different anatomical sites. Importantly, drop‐printing can be performed with basic tools such as pipettes or micropipettes, lowering the barrier to adoption outside specialised microfabrication settings.^7^

## 4 NEUROMODULATION PROOF‐OF‐CONCEPT

Notably, a key finding from this Science study is that drop‐printing enables conformal placement of ultrathin silicon‐based films on soft neural tissues—eliminating the need to redesign such devices into stretchable architectures. In a large‐tissue demonstration, a silicon film was wrapped onto a pig brain surface without fracture, whereas conventional pressing broke the fragile film. This is clinically meaningful because inorganic semiconductors can enable optoelectronic functions and high signal fidelity, yet they are often least tolerant to mechanical stress during placement.^7^, ^8^

In vivo, silicon heterojunction films were drop‐printed onto the exposed sciatic nerve of mice using PBS and stimulated by pulsed near‐infrared light (653 nm). Photostimulation triggered immediate hindlimb movement and compound muscle action potentials, while thermal imaging indicated negligible heating. In rats, films placed on the right primary motor cortex enabled light‐evoked contralateral forelimb movements synchronised with pulsed irradiation, together with stimulation‐evoked neural signals (Figure 1). These findings suggest a potential route toward minimally invasive, light‐controlled neuromodulation without genetic modification—an attractive direction for electroceutical development if durability, safety and system integration can be achieved.^4^, ^7^

Beyond neuromodulation, the core idea—using a transient lubricating interface to convert harmful stretch into controllable sliding—could be valuable across neuroscience and broader clinical medicine. Intraoperative neurophysiology and epilepsy surgery, for example, increasingly rely on rapid placement of high‐density ECoG grids on curved cortical surfaces, where incomplete contact or mechanical damage limits signal quality and mapping precision.^1^, ^3^ Chronic interfaces for the spinal cord, dorsal roots or peripheral nerves also face micromotion and curvature that challenge stable coupling.^2^, ^3^ Outside neuroscience, conformal device–tissue contact is a recurring pain point in dermatology and rehabilitation (skin‐mounted sensors), in soft‐tissue monitoring and wound care, and even in bedside imaging platforms such as conformal ultrasound patches.^9^, ^10^, ^11^ Drop‐printing's ability to wrap fragile films onto complex anatomy therefore points to a general deployment toolkit that could help bring high‐performance microelectronics into clinical environments where geometry, moisture and time are the real constraints.^3^, ^7^

## 5 WHAT CLINICAL TRANSLATION WILL REQUIRE

Clinical translation will require transforming a laboratory technique into a standardised clinical workflow. Three challenges stand out.

First, the method must be compatible with the realities of surgery. Current demonstrations have largely been performed on air‐exposed surfaces under controlled laboratory conditions. Clinical implementation will require reliable operation in wet, bleeding and dynamically moving environments, together with practical protocols for fluid management, evaporation timing and sterile handling.

Second, the interfacial chemistry must be suitable for medical use. Although droplet additives can improve placement precision and adhesion, clinical formulations will need to rely on medical‐grade reagents with validated sterilisation pathways and well‐characterised toxicological profiles.

Third, long‐term reliability remains essential. Reducing deployment stress is only the first step; chronic applications will require stable adhesion under tissue micromotion, durable electrical performance, biostable encapsulation, reliable lead or telemetry integration, and acceptable tissue responses over time, including inflammation and fibrosis.

Reproducibility and standardisation will also be critical, particularly if droplet dynamics and three‐phase contact line (TCL) control are to support automation, multi‐centre studies and regulatory evaluation.

## 6 CONCLUSION

Drop‐printing reframes conformal bioelectronics as a deployment problem that can be solved by stress management rather than by stretchable redesign alone. By combining capillary‐driven wrapping with sliding‐enabled stress release, it offers a gentle route to place fragile, high‐performance films onto living curved tissues with controllable accuracy. If this method can be developed into sterile, reproducible surgical workflows and proven to be reliable in long‐term chronic applications, droplet‐mediated deployment will bridge the longstanding divide between advanced microelectronics and the complex clinical anatomical environments where these technologies are most needed.^3^, ^7^
